# Large-Scale Fabrication of SiC-TiC@C Powders via Modified Molten Salt Shielding Synthesis Technique and Their Effect on the Properties of Al_2_O_3_-MgO Castables

**DOI:** 10.3390/ma16175895

**Published:** 2023-08-29

**Authors:** Yong Li, Yicheng Yin, Jing Chen, Xiaoxu Kang, Shihao Kang, Haoxuan Ma, Shaowei Zhang, Quanli Jia

**Affiliations:** 1 Henan Key Laboratory of High Temperature Functional Ceramics, School of Materials Science and Engineering, Zhengzhou University, Zhengzhou 450001, Chinayinyicheng@naura.com (Y.Y.);; 2 Henan Hongyu New Materials Technology Co., Ltd., Gongyi 451200, China; 3 College of Engineering, Mathematics and Physical Sciences, University of Exeter, Exeter EX4 4QF, UK; s.zhang@exeter.ac.uk

**Keywords:** SiC-TiC coatings, graphite flake, Al_2_O_3_-MgO castables, modified molten salt shielding synthesis technique, water-wettability

## Abstract

Graphite flakes are commonly used to fabricate carbon-based refractories owing to their superior properties, including better corrosion resistance and thermal shock resistance (TSR); unfortunately, their insufficient water-wettability has remarkably hindered their application in castables. Aiming to enhance their water-wettability, a facile and low-cost technique for fabricating carbides coated in graphite was proposed in this work. Firstly, SiC-TiC coated graphite (SiC-TiC@C) powders were prepared via modified molten salt shielding synthesis in an air atmosphere using graphite flake, Si and Ti powders as raw materials and NaCl-KCl as the molten salt shielding medium. Water-wettability and oxidation resistance of SiC-TiC@C powders were significantly improved. Compared to the Al_2_O_3_-MgO castables with graphite flakes, the water demand of the castables with SiC-TiC@C was noticeably decreased from 6.85% to 4.89%, thereby decreasing the apparent porosity of the castables with 5% SiC-TiC@C (from 20.3% to 13%), enhancing the cold strength, hot strength and oxidation resistance of the castables. Such enhancements are ascribed to continuous and crack-free SiC-TiC coatings on graphite surfaces ensuring that the castables have outstanding properties.

## 1. Introduction

Carbon-containing shaped refractories for steel ladle linings have been increasingly replaced by Al_2_O_3_-MgO castables because of their easier installation, monolithic linings, shorter manufacturing cycles, long service life, lower thermal conductivity, etc. [1,2,3]. Recently, secondary refining processes, including higher temperature (>1650 °C), higher stirring intensity (>1 MPa) and longer stirring periods (>1 h), have been conducted in steel ladles, therefore making their service conditions more severe and shortening the service life of ladle linings [4,5,6,7,8]. Therefore, performances, including the slag resistance and TSR of the castable linings in steel ladles, need to be improved [6,7,8,9,10,11]. Several attempts have been made to incorporate graphite flakes in castables because of their good TSR and non-wettability by slag and liquid steel [12,13,14,15]. Unfortunately, their non-wettability and poor dispersity resulted in the higher water demand of the castables, which noticeably deteriorated their properties [12,13,14,15,16,17]. 

To date, some works have reported that the water-wettability of graphite flakes can be significantly enhanced by carbide coatings (SiC, TiC, WC, etc.) on their surfaces via the molten salt method (MSS) in a flowing argon atmosphere [17,18,19,20,21,22,23,24]. An inert atmosphere is required in a conventional MSS method, resulting in difficulty in mass production and high cost for fabricating carbide-coated graphite [21,22,23,24], thereby limiting its application in carbon-containing castables. We found that carbides and carbide-coated graphite including SiC@C and TiC@C can be achieved via a molten salt shielding synthesis (MS^3^) technique in an air atmosphere [25,26,27,28], and as-prepared carbide-coated graphite flakes possessed good water-wettability and oxidation resistance.

Combined with the good wettability of TiC coating and good dispersion of SiC coating, the SiC-TiC-composite-coated graphite may demonstrate good performance [25,26]. In this work, a modified molten salt shielding synthesis (MMS^3^) method without salt encapsulation for fabricating SiC-TiC@C using Si, Ti and graphite flakes in an air atmosphere was proposed, and the effect of SiC-TiC@C on the properties of Al_2_O_3_-MgO castables was also evaluated.

## 2. Materials and Methods

Graphite flakes (C ≥ 97%, <44 μm), Si fine (Si ≥ 98%, <44 μm) and Ti powders (T ≥ 98%, <44 μm) were utilized as starting materials. NaCl and KCl (AR, <100 μm) were used as molten salt medium. Firstly, SiC-TiC@C powders were prepared via a MMS^3^ technique. The molar ratios of metal powders (Si/Ti = 1/1) to graphite were 1/2, 1/4 and 1/8, which were labeled as STG12, STG14 and STG18, respectively, and the mass ratio of raw materials (Si, Ti and graphite) and NaCl-KCl was 1:1. Raw material mixtures and ethanol were mixed and dried at 110 °C, and were pressed into φ 36 × 36 mm cylinder specimens. Thereafter, the specimens were placed into an alumina crucible and covered by NaCl-KCl, and then the crucible was heated to 1150–1300 °C for 3 h in an air atmosphere. After cooling, the solidified samples were dissolved by deionized water and washed repeatedly; finally, the SiC-TiC@C powders were acquired via filtrating and drying. The detailed processes were similar to our previous works [25,26,27,28].

Al_2_O_3_-MgO castables without and with graphite flakes, STG14 and STG12, were designed and denoted as N-C, G-C, STG-14C and STG-12C, respectively. The castables, after mixing with water, were casted in bar-shaped samples (25 mm × 25 mm × 150 mm) under vibration, and then these samples were cured at room temperature for 24 h, and they were dried at 110 °C for 24 h, and treated at 1000 °C and 1600 °C for 3 h under a reducing atmosphere. Physical properties, such as the apparent porosity (AP), cold modulus of rupture (CMOR) and permanent linear change (PLC) of the castables, were characterized. Phase compositions and microstructures of resultant powders were evaluated by X-ray diffraction (XRD, Philiphs Xpert Pro, Almelo, The Netherlands) and scanning electron microscopy (SEM, EVO HD15, Zeiss, Jena, Germany). Water dispersity of graphite was measured via sedimentation experiment recording by digital camera. Oxidization behavior of SiC-TiC@C powders was identified using thermogravimetric (TG) analysis.

## 3. Results

**Figure 1:**
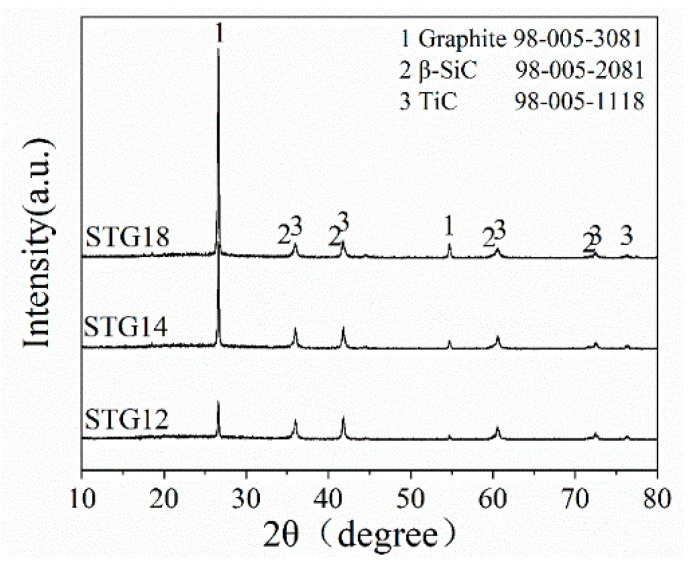
XRD patterns of STG12, STG14 and STG18 fired at 1300 °C for 3 h.

Figure 1 shows the XRD patterns of as-fabricated SiC-TiC@C powders. As shown in Figure 1, the main phases of STG products are graphite, β-SiC and TiC, no Ti, Si and oxide phases. Furthermore, the height of β-SiC and TiC peaks shows almost no change, and the peak height of graphite increases significantly with augmenting of Si + Ti/C ratio from 1/2 to 1/8. These results demonstrate that SiC-TiC@C powders are successfully synthesized via a MMS^3^ technique in an air atmosphere.

**Figure 2:**
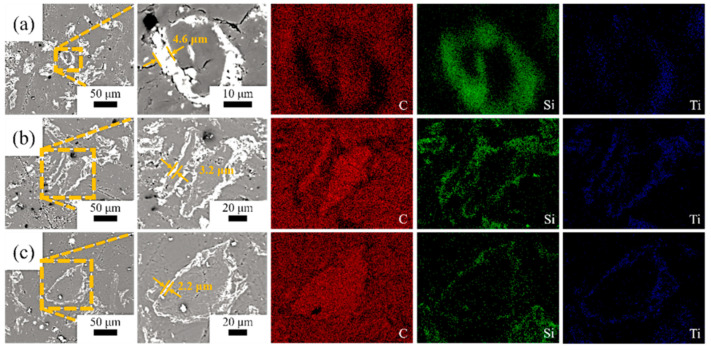
SEM photographs and EDS mapping of (**a**) STG12, (**b**) STG14 and (**c**) STG18.

Figure 2 shows the morphologies of the SiC-TiC@C powders. It can be seen that the dark graphite flake is partially or completely covered by bright white SiC-TiC composite coating. When the Si + Ti/C ratio augments from 1/8 to 1/2, the average coating thickness obviously increases from 2.2 µm to 4.6 µm. More coating particles can be observed in samples STG12 and STG14. EDS mappings show that the sample is mainly comprised of C, Si and Ti, and the shell layer is comprised of Si, Ti and C, indicating that the shell is formed of a SiC and TiC composite coating. EDS and XRD (Figure 1) confirm that the SiC-TiC layer is formed on graphite flake, and indicated core–shell characteristics.

**Figure 3:**
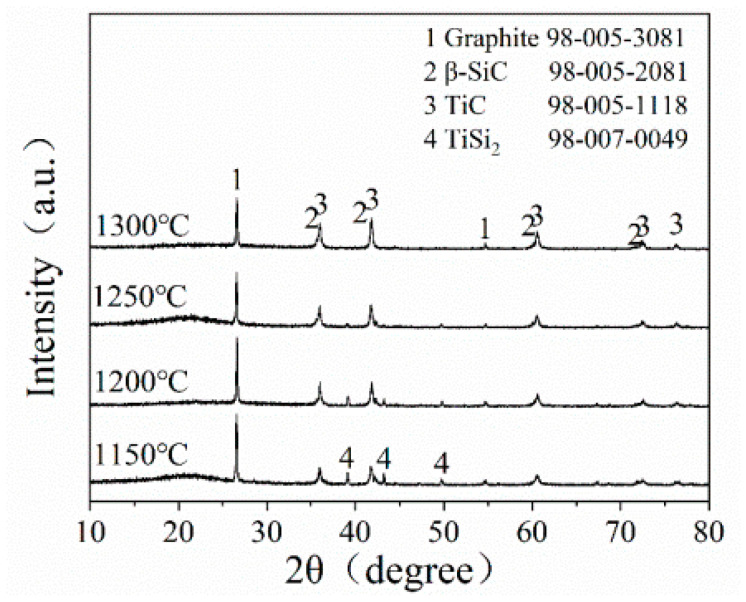
XRD patterns of STG12 heat-treated at 1150~1300 °C.

Figure 3 presents the XRD pattern of STG12 sample after firing at 1150–1300 °C for 3 h. At 1150 °C, the main phases of STG products are graphite, TiC and TiSi_2_. On raising the temperature to 1200 °C, the crystal phases do not change, while the peak height of TiSi_2_ slightly decreases. At 1250 °C, TiSi_2_ almost disappears, and SiC peaks can be observed. At 1300 °C, TiC, SiC and graphite are detected, indicating that SiC-TiC@C can be obtained at 1300 °C. These results are similar to that of SiC-coated graphite flakes via a MMS^3^ technique in an air atmosphere [25,26,27]. As seen in Figure 3, the sample fired at 1150–1200 °C shows no Si peaks due to Si reacting with Ti to form TiSi_2_ in a molten salt medium; the reasons will be discussed in the following section.

**Figure 4:**
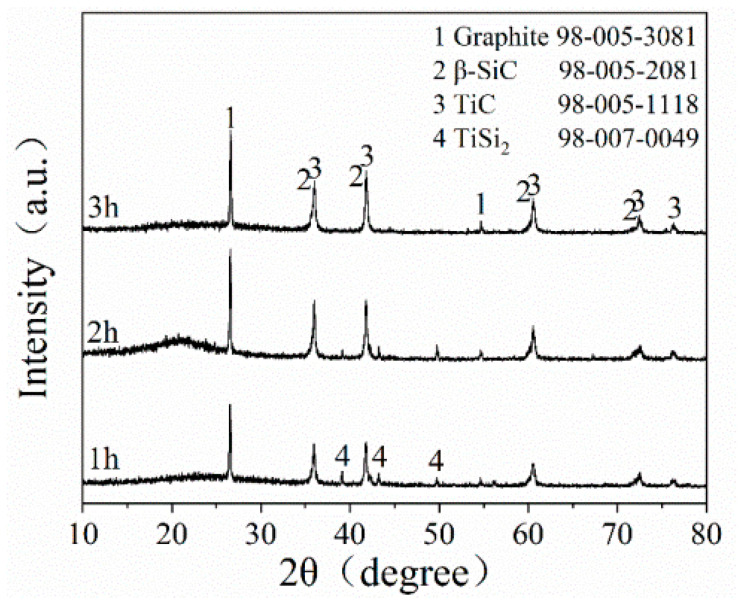
XRD pattern of STG12 fired at 1300 °C for 1–3 h.

Figure 4 depicts the XRD patterns of STG12 sample synthesized at 1300 °C for 1–3 h. The main phases of STG products are graphite, TiC and SiC, and minor TiSi_2_ peaks are also detected at 1300 °C for 1 h. When increasing the dwelling time from 1 h to 3 h, the intensity of SiC and TiC peaks increases obviously, and the TiSi_2_ peaks disappeare, indicating that longer holding time is beneficial to the reaction of intermediate phase (TiSi_2_) and graphite to form TiC and SiC.

**Figure 5:**
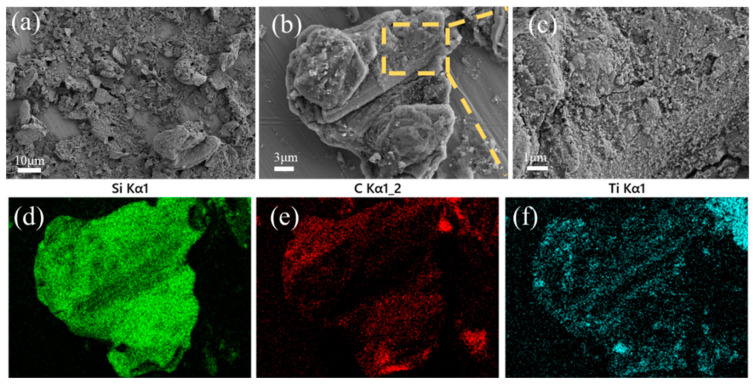
(**a**–**c**) SEM images and (**d**–**f**) EDS mappings of STG12 fired at 1300 °C.

Figure 5 demonstrates SEM images and EDS mappings of sample STG12. The uneven surface of graphite flakes is an indication of in situ formed SiC-TiC coatings. Heterogeneously distributed particles are found on their surfaces, and they completely cover the surface of graphite with sizes of about several hundred nanometers. EDS mappings show that Ti, Si and C spectra are found (Figure 5d–f), showing that a TiC-SiC layer is formed on the graphite’s surface. Herein, rough but uniform and crack-free coatings contributed to improving the water-wettability of coated graphite.

**Figure 6:**
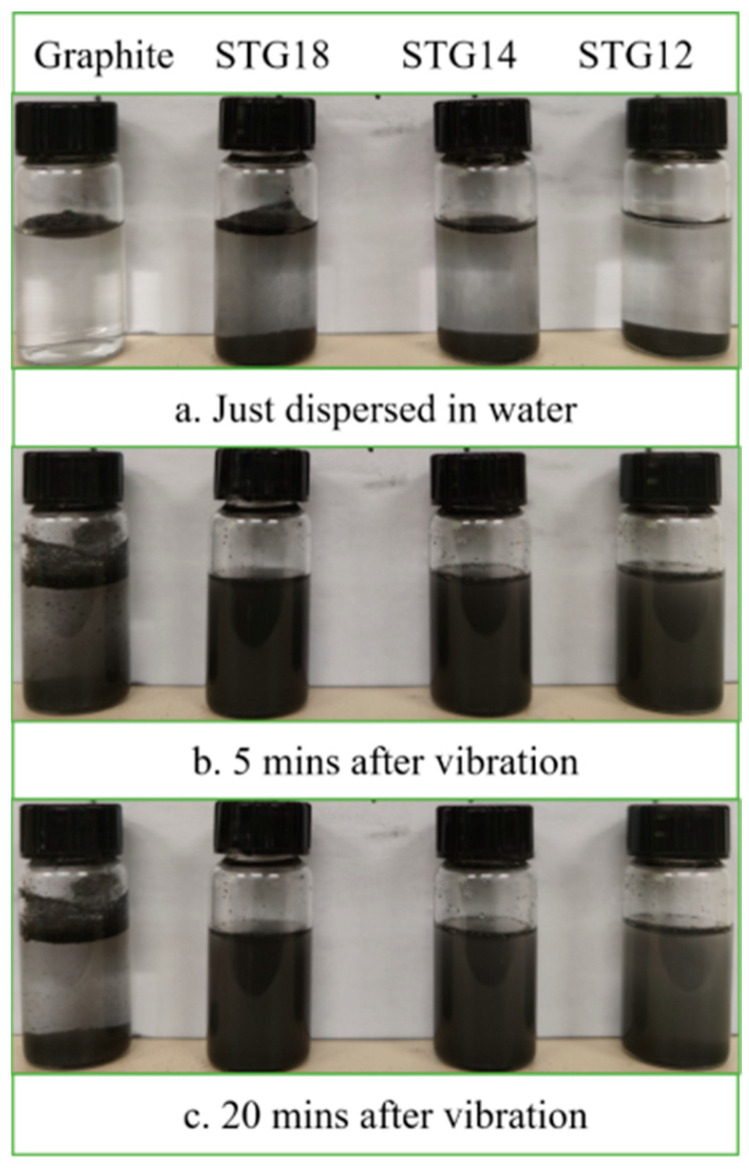
Photographs of uncoated and coated graphite suspension.

Water-wettability of the samples was verified via a sedimentation test and presented in Figure 6. As shown in Figure 6, all of the graphite flakes are floating on the surface of the water, indicating that their water-wettability is very poor after just being dispersed in water. In contrast, some SiC-TiC@C particles immediately disperse into water. After vibration, under the action of gravity, two layers of the graphite particles can be seen after 5 min and 20 min; some particles float on the surface of the water, while others sink on the bottom. The SiC-TiC@C particles evenly disperse into water and form a suspension after placing for 20 min. Sedimentation test reveals that the water-wettability of the coated graphite is significantly improved.

**Figure 7:**
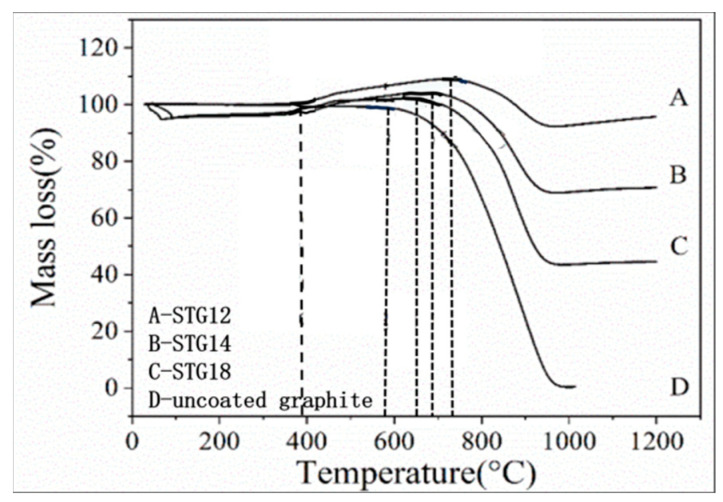
TG curves of uncoated graphite, STG12, STG14 and STG18.

Figure 7 shows the TG curves of graphite powders. As for uncoated graphite, the initial oxidation temperature is 580 °C. As for coated graphite, on increasing the temperature from 390 °C to 650–740 °C, TG curves indicate that the mass gain of STG18, STG14 and STG12 tends to increase, the maximum weight gain is 1.31%, 3.98% and 9.24% and the corresponding oxidation temperature is 650 °C, 690 °C and 740 °C, respectively, for STG18, STG14 and STG12, which is much lower than that of TiC-coated graphite [28], but more than SiC-coated graphite [25]. At 1000 °C, the weight loss of the uncoated material is 99%, and 55%, 29% and 4%, respectively, for STG18, STG14 and STG12. TG curves, XRD (Figure 1) and SEM images (Figure 2 and Figure 5) show that the thickness increase of coating quantity can significantly improve the oxidation resistance of graphite flakes, which is conducive to decreasing the contact content between oxygen atoms and graphite; furthermore, TiO_2_ and SiO_2_ are formed by oxidization of SiC and TiC particles, which also can deter graphite from being oxidized by oxygen, thereby enhancing oxidation resistance of the graphite.

**Figure 8:**
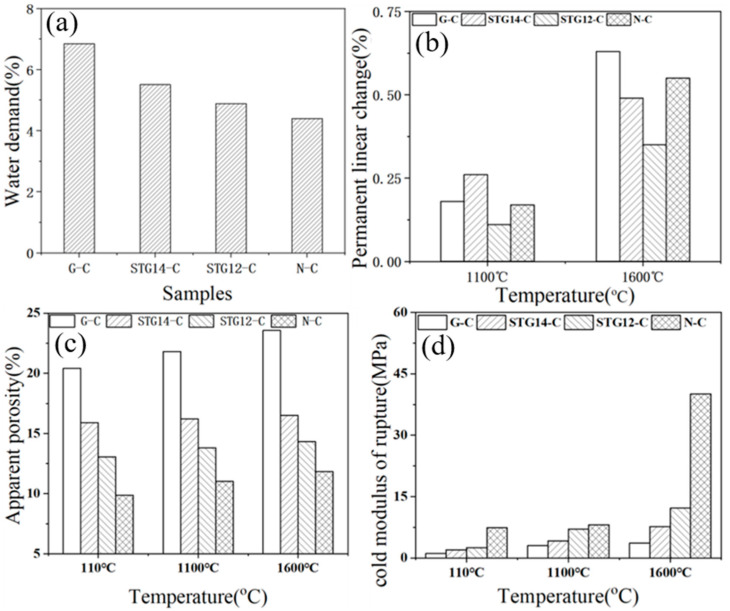
Water demand (**a**), PLC (**b**), AP (**c**) and CMOR (**d**) of the castables.

**Figure 9:**
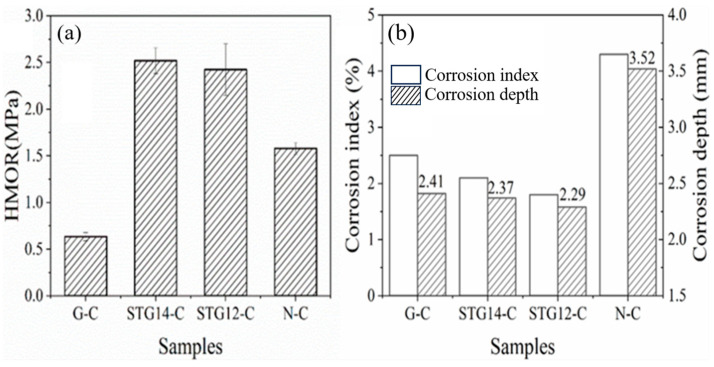
(**a**) HMOR and (**b**) corrosion index and depth of the castables.

The above-mentioned results show that SiC-TiC composite coatings can be generated on the surfaces of graphite flakes using MMS^3^, which greatly improves their wettability and oxidation resistance. To demonstrate the performance of Al_2_O_3_–MgO castables containing SiC-TiC@C powders, the effect of SiC-TiC@C on the properties of the castables was further investigated and presented in Figure 8 and Figure 9.

Water demand (Figure 8a) of the castables is 6.85%, 5.51%, 4.89% and 4.4%, respectively, for samples G-C, TG-14C, TG-12C and N-C, which is lower than the castables containing SiC@C (5.25%) [25], further demonstrating that SiC-TiC@C powders can further reduce the water demand and improve the water-wettability of the Al_2_O_3_–MgO castables. PLC value (Figure 8b) of the samples with SiC-TiC@C powder addition after firing at 1600 °C decreases from 0.64% to 0.35%. As shown in Figure 8c, the AP value of sample G-C increases from 20.3% to 23.56% after heat-treating at 110 °C, 1100 °C and 1600 °C, which is 15.42%, 15.83% for sample STG-14C, and 13.97%, 14.01% for sample STG-12C. CMOR values (Figure 8d) of the samples after heat treatment at 110 °C and 1600 °C are also noticeably increased from 1.0 MPa to 3.5 MPa, and from 3.64 MPa to 12.16 MPa, respectively. Those results indicated that physical properties of samples with SiC-TiC@C powders are obviously enhanced in comparison with the sample with as-received graphite flakes, which is ascribed to SiC-TiC@C possessing good water-wettability and sinterability, thereby decreasing water demand and improving their physical properties.

HMOR testing of the sample at 1400 °C in the carbon-embedded condition is shown in Figure 9a. The results depict that HMOR of STG14-C and STG12-C (2.52 MPa, 2.43 MPa) is much higher than that of G-C (0.64 MPa), and also higher than that of N-C (1.58 MPa), indicating that the HMOR of Al_2_O_3_-MgO-C castables with SiC-TiC@C addition greatly increases. This may be related to the castables containing certain amounts of TiC and SiC, which is beneficial to improving the hot strength of Al_2_O_3_-MgO castables because the castables have little linear expansion, and fewer low-melting-point phases in the matrices.

The corrosion depth and corrosion index was calculated by IPP 6.0 software and shown in Figure 9b. It can be seen that the corrosion index is 4.3%, 2.5%, 2.1% and 1.8%, respectively, and the corrosion depth is 2.41 mm, 2.09 mm and 2.13 mm, respectively, for samples G-C, TG14-C and TG12-C, showing a significant reduction compared to that of sample N-C without graphite (3.52 mm). This indicates that the castables with coated graphite (STG-14C, STG-12C) possessmuch better slag resistance than that of samples G-C and N-C. This is due to the slag penetration being decreased by graphite powders because of their poor wettability to slag.

## 4. Discussion

**Figure 10:**
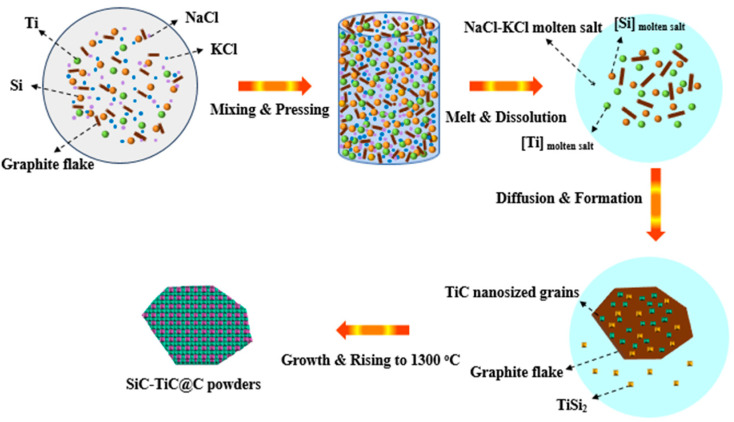
Schematic diagram for the growth of SiC-TiC@C via MMS^3^ route.

TiC has been formed at >800 °C and SiC can be formed at 1300 °C (Figure 4). XRD (Figure 1) and SEM analysis (Figure 4 and Figure 5) confirm that continuous and crack-free SiC-TiC coatings covered the graphite after firing at 1300 °C in an air atmosphere via MMS^3^ technique. The reasons and growth mechanism are as follows. To clearly demonstrate the growth mechanism of SiC-TiC coating, a schematic diagram is presented in Figure 10. Firstly, as the temperature exceeds the melting point of NaCl-KCl (about 663 °C), molten salt medium can provide a barrier to protect the starting materials from oxidation during the heating process in an air atmosphere, and partial Ti and Si particles can dissolve into salt melts and develop nanosized grains via the dissolution–diffusion mechanism, which has been verified in refs [25,28,29,30,31,32,33]. Secondly, the formation of TiC via reacting of Ti and graphite is also facilitated by salt melts. Considering that Si disappears, TiSix is detected, which due to Si reacts with Ti to form the Ti–Si eutectic phase [21,34,35]. On rising the temperature to 1300 °C, TiSix can react with graphite to generate TiC and SiC via the template growth mechanism [21,22,25,26,28,29]. On rising the reaction temperature and dwelling time, the formation of TiC and SiC continuously occurs until TiSix is completely consumed (confirmed by Figure 3 and Figure 4). Finally, nanosized SiC-TiC particles are generated on the graphite surface and develop homogenous and crack-free coatings.

The formation mechanism of SiC-TiC coatings is different to that of SiC- or TiC-coated graphite. As reported [25,26,28], TiC coating can be completely formed at 900 °C, and SiC coating can be completely formed at 1350 °C; however, no Si was detected at 1150–1250 °C in this work, which is due to Ti reacting with Si to form the TiSi_2_ alloy [21,34,35]. These contribute to generating nanosized SiC-TiC coatings on graphite surfaces (Figure 4 and Figure 5), thereby creating SiC-TiC@C powders possess better performance compared to the as-received graphite flakes (in Figure 8 and Figure 9). Using combined TG, XRD and SEM analysis, upon increasing thickness of coating on graphite flakes, it can be seen that the water demand of the sample STG12-C is noticeably reduced, thereby reducing AP, enhancing CMOR and HMOR, oxidation resistance and slag corrosion resistance of Al_2_O_3_-MgO castables.

## 5. Conclusions

A facile method for fabricating SiC-TiC@C in an air atmosphere was proposed in this manuscript. Results show that TiC-SiC-coated graphite was obtained by reacting Ti, Si and graphite in a NaCl and KCl mixture via the MMS^3^ route in an air atmosphere at 1300 °C. The as-prepared nano-sized composite coatings were continuous and crack-free, making as-fabricated SiC-TiC@C powders possesse good performance, including water-wettability and oxidation resistance. Water demand of Al_2_O_3_-MgO castables with SiC-TiC@C addition was noticeably decreased from 6.89% to 4.89%, apparent porosity significantly reduced from 20.3% to 13%, and cold strength, hot strength, oxidation resistance and slag resistance of Al_2_O_3_-MgO castables were noticeably enhanced in comparison with the castables with graphite flakes. The reasons may be ascribed to dense SiC-TiC coatings endowing graphite flakes with good water-wettability, sinterability and oxidation resistance, thereby decreasing water demand and improving the physical properties, hot strength and corrosion resistance of the castables.

## Data Availability

The data presented in this study are available on request from the corresponding author.
